# Optimising the Delphi survey method during core set development: The impact of summarised feedback on stakeholders’ prioritisation of core data items and consensus

**DOI:** 10.1371/journal.pone.0348136

**Published:** 2026-06-10

**Authors:** Katy A. Chalmers, Karen Coulman, Jane M. Blazeby, John Dixon, Lilian Kow, Ronald Liem, Dimitri J. Pournaras, Johan Ottosson, Richard Welbourn, Wendy Brown, Kerry N. L. Avery

**Affiliations:** 1 National Institute for Health and Care Research Bristol Biomedical Research Centre, University Hospitals Bristol and Weston NHS Foundation Trust and University of Bristol, Bristol, United Kingdom; 2 Bristol Centre for Surgical Research, University of Bristol, Bristol, United Kingdom; 3 Obesity and Bariatric Surgery Service, North Bristol NHS Trust, Bristol, United Kingdom; 4 Iverson Health Innovation Research Institute, Swinburne University of Technology, Melbourne, Australia; 5 College of Medicine and Public Health, Flinders University, Adelaide, Australia; 6 Department of Surgery, Groene Hart Hospital, Gouda, The Netherlands; 7 School of Medical Sciences, Örebro University, Örebro, Sweden; 8 Department of Upper GI and Bariatric Surgery, Somerset NHS Foundation Trust, Taunton, United Kingdom; 9 Department of Surgery, Monash University, Melbourne, Australia; IESEG School of Management, FRANCE

## Abstract

**Background:**

Core outcome sets are an established method for standardising the collection, measurement and reporting of treatment outcomes in effectiveness trials. Using Delphi survey methodology, core sets are developed by prioritising and re-prioritising data items facilitated by provision of feedback of other stakeholders’ responses. It is unknown how best to provide feedback to ensure that it influences the re-prioritisation of items effectively. This study examined whether informing participants of the top-rated items from the previous survey round may influence the re-prioritisation of data items in a subsequent survey round during the development of core data sets.

**Methods:**

This study was nested in the development of a registry core data set. In round two of the Delphi survey, participants were randomised to receive ‘standard’ or ‘enhanced’ instructions. ‘Enhanced’ instructions included summarised data of the top five data items scored by participants in the previous survey round) in addition to standard feedback (the median round 1 score per item). Items scored 7–9 by ≥70% of participants in round 2 were considered ‘prioritised’. Concordant/discordant items were determined and extent of agreement between groups calculated (kappa statistics).

**Results:**

Both groups prioritised a larger number of items in round 2 than in round 1 and there was little difference in the percentage of respondents prioritising the ‘Top 5’ items in round 2 (mean change in prioritisation of Top 5 items for all four core sets combined – 2.3% increase in standard group and 3.2% increase in enhanced group). Overall agreement in data items prioritised by both groups improved in round 2 (discordant items – 11% in round 1 and 4% in round 2).

**Conclusion:**

Providing participants with additional feedback during the process of item prioritisation did not promote prioritisation of items during development of a core set. In the development of health core sets, where often many items are prioritised, further work to determine how to clearly and optimally communicatee feedback in a manner that promotes consensus effectively is required. Specifically, qualitative work with relevant stakeholders, exploring and clarifying the concepts of prioritisation and consensus, is warranted.

## Introduction

Comparing and synthesising data across studies is fundamental to answering important clinical questions about health and clinical care. The ability to compare and combine data is, however, dependent on the compatibility of data. The development and use of a core data set, such as a core outcome set (COS), is one established approach to improve data synthesis [1]. COS are being increasingly developed for use in comparative effectiveness trials but are also important to standardise reporting in other contexts, such as disease- or treatment-specific registries. As such, there is growing interest in core set development methodology to optimise the efficiency and effectiveness of the development process and Delphi surveys are commonly used to prioritise core set items [2,3].

The Delphi survey method uses a simple framework in which researchers have flexibility in the methods used to seek consensus on a core set [4]. A key feature of this process is the provision of feedback to participants in subsequent survey rounds, which enables other participants’ opinions to be considered before re-prioritisation of items. Whilst ‘describe how participants receive feedback’ is an item on a checklist to standardise COS development [3], there is no agreed format of how it should be presented. Previous studies have shown the importance of feedback presentation, since patients and healthcare professionals prioritise different types of outcomes [5] and providing feedback from individual stakeholder groups may improve consensus between groups and agreement in items prioritised [6]. But how stakeholders interpret and utilise feedback, is unknown. Most Delphi studies use a Likert scale to score items [4,7] and therefore feedback is commonly presented as a summary statistic such as a mean or median value, with or without an indicator of range (e.g., standard deviation, inter-quartile range) [4]. Several published studies have noted that rating scales, such as Likert, lead to less variation in scores across items as repetition of scores is allowed (compared to ranking) [8] and that health Delphi studies tend to prioritise more items due to their perceived importance [8]. Together, in a health Delphi, this results in many items being scored highly and does not allow differentiation between the items considered the most important; a problem when the purpose of Delphi surveys is to reduce the number of items prioritised in each round. Given the increasing interest in the development of core sets in health service research, it seems pertinent to identify ways to improve the efficiency of prioritisation and consensus.

The aim of this nested randomised controlled trial (RCT) was to explore whether the provision of summarised feedback in addition to usual feedback (enhanced instructions), promoted the prioritisation of items compared to those receiving usual feedback only (standard feedback). This was explored within a Delphi survey for the prioritisation of items in a core data set for an obesity surgery registry.

## Methods

### Design

This was a prospective, parallel-group RCT nested within an ongoing study to develop core data sets for an international bariatric surgery registry [9]. The core data set development study comprised 3 steps: (i) identification of a long list of data items; (ii) the Delphi survey and (iii) the consensus meeting to finalise the core data sets. This RCT was nested within the Delphi survey (step ii). Methods for the original core data sets’ development study have been reported previously [9]. Further details of the Delphi survey step are briefly outlined below to provide relevant context to the methodological RCT.

### Participants

All members of the International Federation of Surgery for Obesity and Metabolic Disorders (IFSO) were sent an email from the IFSO president, explaining the core data set project and inviting them to take part in the survey, if they wished. IFSO comprises a wide range of healthcare professionals including bariatric surgeons, psychologists, dietitians, bariatric physicians, and specialist nurses. Since this core data set did not focus on patient-reported outcomes, patients were not included as participants in the consensus process, however an international PPI group of people who had undergone bariatric surgery advised on the project.

Ethical approval for the study was granted by the Faculty of Health Sciences Research Ethics Committee (FREC) (ref. 116384) at the University of Bristol, UK. In line with other Delphi surveys completed by our group, consent was implied by completion of the survey; this approach was discussed with and approved by the FREC. Participants were not made aware that they were participating in a methodological study and were to be randomised to either standard or enhanced instructions in the round 2 survey since this may have biased the results. All procedures performed in studies involving human participants were in accordance with the ethical standards of the institutional and/or national research committee and with the 1964 Helsinki declaration and its later amendments or comparable ethical standards.

### Delphi survey

#### Survey development.

A comprehensive list of potentially relevant items for the core sets was identified from earlier work, including a previous COS for bariatric surgery trials [5], a bariatric registry data dictionary project [10], systematic literature reviews and patient and public involvement group discussions. Items from each source were collated into a single long list where duplicates were removed and overlapping themes combined. The study team, comprising methodologists and health professionals, grouped the items into broader domains which reflected the different phases of data collection within bariatric registries. These items were included in a 97-item questionnaire divided into sections which would go on to form the four core data sets – 1. baseline information, 2. effectiveness outcomes, 3. surgical procedure information and 4. potential complications and side-effects of surgery. Free text boxes were included at the end of each section to enable stakeholders to propose new items. The electronic questionnaire was hosted on REDCap data capture software [11,12] and sent to all IFSO members via email.

#### Prioritisation of items.

In round 1 of the Delphi survey, all participants received identical surveys. They were asked to rate the importance of each item for inclusion in the final core data sets on a Likert scale of 1–9. A score of 7–9 indicated an item of critical importance, 4–6 an item important but not critical, and 1–3 an item of limited importance. Median scores for each item were calculated for all participants combined and for each stakeholder group (bariatric surgeons, dietitians, specialist nurses, psychologists, other). For round 2, in line with COMET guidelines [4], all data items were retained, regardless of their scoring in round 1. All responding participants received the same survey as in round 1 plus anonymised feedback for each item detailing their own score, their peer group’s median score, the other stakeholder groups (combined) median score and the overall median score. In addition to the feedback scores, patients were randomised to receive either ‘standard’ or ‘enhanced’ instructions to guide them in re-rating the items in round 2 (see ‘Randomisation’ below).

The percentage of participants rating each item 7–9 (critically important) after round 2 was calculated. In line with previous core sets [13,14] and COS guidance documents [3,13–16], consensus to prioritise an item was defined, *a priori*, as being achieved if at least 70% of responding participants scored the item 7–9.

Items were categorised into three groups to facilitate discussion and voting on the final core set at the consensus meeting. These were: (1) items rated 7–9 by ≥95% participants to be ratified ‘in’ if no objections; (2) items rated 7–9 by 70–95% of all participants to be discussed in small groups and voted on by the whole group; and (3) items rated 7–9 by <70% of all participants to be ratified ‘out’ if no objections. Full details of the consensus meeting discussions and voting criteria have been described previously [9]. The final core data sets comprised 12 items [9].

#### Instructions.

**Fig 1:**
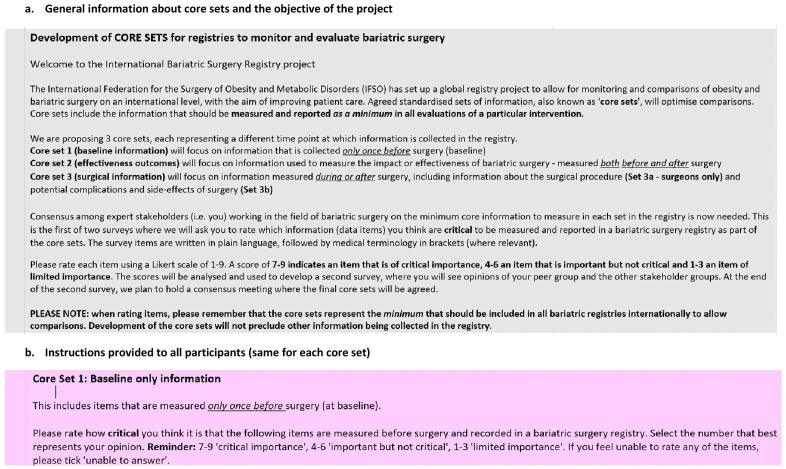
Information and instructions presented to all participants to guide them in the completion of the questions in the round 1 survey.

**Fig 2:**
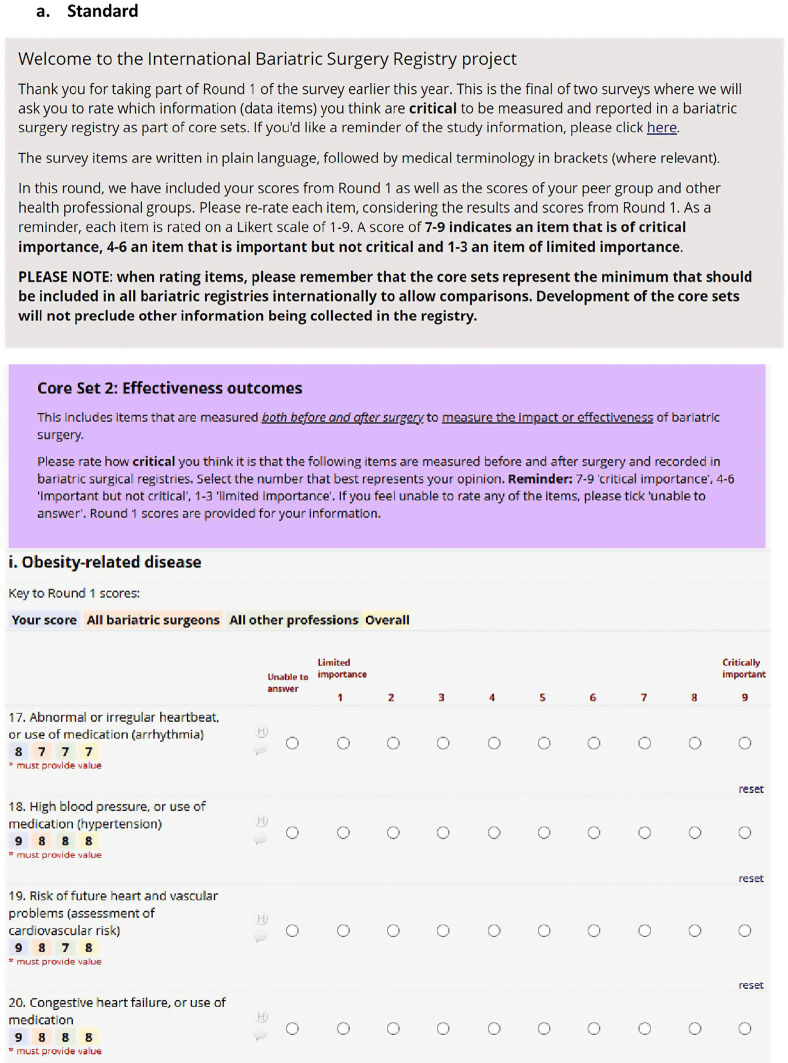
Information about the feedback (results) provided and instructions for how to re-rate data items in round 2. Participants randomised to either standard (a) or enhanced (b) instructions in the round 2 survey.

In the round 1 survey, all participants received identical information about core sets and instructions on how to complete the survey (Fig 1). For the round 2 survey, two types of instructions (standard and enhanced) were developed (Fig 2). Standard instructions emphasised the provision of feedback from peer groups and other healthcare professionals and offered simple guidance on how to use the feedback presented when re-rating the data items for round 2 (Fig 2a). This is similar to that used in previous Delphi surveys to develop COS [13,17]. The aim of the enhanced instructions was to provide participants with summarised data of the ‘Top 5’ data items for each core set in order to facilitate re-rating data items in round 2. The Top 5 items were the five items prioritised (scored 7–9) by the highest percentage of participants in round 1. As with standard instructions, the introductory information emphasised the provision of feedback from peer groups and other healthcare professional groups but in addition, highlighted that the Top 5 data items for each core set were also provided (Fig 2b). For each of the four core sets, the Top 5 data items for each of the four sections were displayed (Fig 2b) with instructions on how to re-rate items.

### Randomisation

Participants who answered at least one question in round 1 of the Delphi survey, were randomised to receive either the standard or enhanced instructions in round 2. For those participants who had more than one identity number due to multiple openings of the survey, the most complete entry was included in the randomisation and the others, excluded. If completion rates were the same for all entries by one individual, the first entry was included. Participants with an odd numbered record ID were assigned standard instructions and those with an even numbered record ID, enhanced instructions. Randomisation was performed by the REDCap administrator using REDCap software [11,12]. Because of non-responders and participants with more than one ID number, randomisation to each instruction group was not strictly 1:1.

### Sample size

This RCT was nested within an ongoing Delphi survey to develop a COS and, as such, the maximum possible sample size was restricted by the size of the Delphi survey. This approach reflects similar methodological studies nested within COS development studies where hypothesis testing is primarily exploratory.

### Data analyses

The objective of this study was to explore the effects of instruction type (standard or enhanced) on the prioritisation of core items across two Delphi survey rounds. As such, only respondents who answered at least one question in both rounds 1 and 2 were included in the analyses. To establish whether respondents altered their responses in round 2 from round 1, data from round 1 respondents were grouped according to which set of instructions they received in round 2.

#### Response rates.

Response rates were calculated for each version of the survey (standard or enhanced) with the total number of participants per randomisation group as the denominator.

#### Prioritisation of items.

The number and percentage of items prioritised (rated 7–9 by ≥70% of participants) by each group was calculated for both rounds.

#### Agreement in items prioritised.

The number and percentage of concordant items (items that both groups did or did not prioritise) and discordant items (items that only one group prioritised) prioritised in each round was also calculated and the level of agreement between groups determined using kappa statistic (κ) [18]. The level of agreement was classified as poor (κ ≤ 0.0), slight (κ 0.01–0.20), fair (κ 0.21–0.40), moderate (κ 0.41–0.60), substantial (κ 0.61–0.80), or almost perfect or perfect (κ 0.81–1.0) [18]. All statistical analyses were performed in Stata version 17 [19].

#### Differences in prioritisation of ‘Top 5’ items.

The differences between groups in the percentage of participants prioritising round 1 ‘Top 5’ items (i.e., the 5 items for which the highest proportion of participants in each group scored the item as 7–9 in round 1) was calculated by subtracting the item scores in round 1 from round 2. A positive number signified an increase in the number of participants prioritising the item.

## Results

### Response rates and stakeholder characteristics of randomisation groups

All members of IFSO were invited by email to take part in the survey. The survey link was live for 6 months (April-September 2021) to allow a reasonable time for as many people as possible to complete the round 1 and round 2 surveys. A reminder was sent to all IFSO members in July 2021. In round 1, 272 participants answered at least one question; 260/272 (96%) participants completed section 1, 198 (73%) completed section 2 and 192 (71%) completed the whole survey*.* The least number of questions answered was three, by five people*.* All 272 round 1 respondents were randomised to receive either standard (n = 131, 48%) or enhanced (n = 141, 52%) instructions in round 2. In round 2, 123 participants (57 (44%) receiving standard instructions and 66 (47%) receiving enhanced instructions), answered at least one question (response rate of 45%). The majority (122/123 (99%)) completed section 1, 119 (97%) completed section 2 and 114 (93%) completed the whole survey. The least number of questions answered was 12, by one person. There was no difference in round 2 response rates between the two instruction groups. Details of participants’ characteristics, who answered both survey rounds, are presented in Table 1. Most respondents were surgeons, with a higher percentage of surgeons responding in the standard instruction group than the enhanced group (60% vs 52%). Dietitians were also well represented although similarly across both groups (21% standard vs 24% enhanced). The distribution of specialities in this sub-study, was broadly similar to all participants that completed round 1 [9]. Experience was similar between the groups with 60% of standard instructions and 59% enhanced group having >10 years’ experience. Respondents represented 35 countries with around one quarter from the United Kingdom.

**Table 1:** Details of participants completing both rounds 1 and 2 for each instruction group.

Category	Sub-category	Standard instructions (n = 57) n (%)	Enhanced instructions (n = 66) n (%)
**Profession**	*Surgeon*	34 (60)	34 (52)
	*Bariatric physician*	3 (5)	2 (3)
	*Dietitian*	12 (21)	16 (24)
	*Specialist nurse*	4 (7)	3 (5)
	*Psychologist*	3 (5)	4 (6)
	*Other*	1 (2)	7 (11)
**Experience**	*<5 years*	7 (12)	11 (17)
	*5-10 years*	16 (28)	16 (24)
	*>10 years*	34 (60)	39 (59)
**Country** *(5 most common)*	*United Kingdom*	15 (26)	15 (23)
	*Australia*	5 (9)	9 (14)
	*Netherlands*	4 (7)	9 (14)
	*Brazil*	2 (4)	5 (8)
	*Italy*	3 (5)	2 (3)

### Prioritisation of items

The number of items prioritised (scored 7–9 by ≥70%) by participants increased in both groups between rounds. The standard instructions group prioritised 58 (60%) items in round 1 and 70 (72%) in round 2. These additional 12 items were the result of thirteen further items being prioritised and one item that was prioritised in round 1, not being prioritised in round 2 (Table 2). The enhanced instructions group prioritised 53 items (55%) in round 1 and 66 (68%) in round 2–13 extra items. Differences in the items prioritised (scored 7–9 by ≥70% stakeholders) by at least one group in round 1, 2, or both are shown in Table 2. Items not prioritised by any group in either round are shown in Supplementary Table 1, and are also available in the original publication of the main study [9].

**Table 2:** Items prioritised (scored 7-9 by ≥70% stakeholders) by at least one group in at least one round.

Core set	Item	Standard instructions (% scoring 7–9)		Enhanced instructions (% scoring 7–9)	
		Round 1	Round 2	Round 1	Round 2
1 (n = 9)	History of previous types of bariatric surgery	96.5	96.5	96.8	95.4
	Duration of type 2 diabetes	89.5	93.0	73.4	83.1
	Height of the patient	82.5	86.0	77.8	90.8
	Other medical conditions not directly related to obesity, e.g., type 1 diabetes	80.7	84.2	64.1*^1^	84.6^2^
	Age of the patient	82.5	80.7	85.9	87.7
	Sex of the patient	71.9	77.2	71.9	76.9
	Details about pre-surgery weight loss	68.4	75.0	66.7	67.7*
	Details of what members of the MDT involved	70.2	71.4	66.2*	69.2*
	Details of previous weight loss programs	70.2	69.6^3^	58.7*	56.9
2 (n = 23)	Type 2 diabetes status	94.4	98.1	96.4	96.9
	Medication for Type 2 diabetes	92.6	96.3	92.6	92.2
	Addictive behaviours, e.g., alcohol, gambling, illicit drugs	76.9	96.3	76.4	92.3
	Weight	96.2	96.3	96.2	96.9
	Problems with breathing during sleep (obstructive sleep apnoea)	88.5	92.6	88.7	95.3
	Suicidal thoughts	80.8	92.6	72.7	89.2
	Binge eating	86.5	92.6	85.2	87.7
	Depression, or use of medication	78.8	92.6	79.6	92.3
	Alcohol intake	81.6	92.6	75.0	90.8
	Congestive heart failure, or use of medication	78.0	92.5	71.2	82.8
	Long standing acid reflux, or use of medication (gastro-esophageal reflux or GERD)	84.9	92.5	83.3	95.3
	Smoking status	72.0	90.7	76.9	90.6
	High blood pressure, or use of medication (hypertension)	76.5	88.9	80.4	89.1
	Obesity-related liver disease, e.g., non-alcoholic fatty liver disease	75.5	86.8	81.1	90.6
	Elevated fat and cholesterol in the blood, or use of medication (dyslipidemia)	79.2	85.2	73.1	81.3
	Feelings towards one’s body shape or appearance (body dysmorphia/dysmorphic disorder)	68.6	83.3	59.3	73.8
	Abnormal or irregular heartbeat, or use of medication (arrhythmia)	53.1	79.6	69.1	76.6
	Risk of future heart and vascular problems (assessment of cardiovascular risk)	74.5	79.2	59.3*	81.3
	Male or female reproductive function, e.g., polycystic ovary syndrome, infertility (reproductive dysfunction)	66.0	77.8	71.2*	73.8
	Use of weight loss medication	67.3	77.4	80.4*	73.4
	How well the pancreas produces insulin (ß-cell function)	59.2	73.6	49.0	54.0*
	Body shape, e.g., waist and hip measurements	57.7	72.2	59.6	70.3
	Joint disease, or use of medication, or being considered for joint replacement	64.7	70.4	71.7*	78.1
3a (n = 14)	Type/make of device (including band and balloon, adjustable or non-adjustable)	76.9	86.2	72.0	88.2
	Method of balloon placement, e.g., swallowed or endoscopically placed	66.7	85.7	69.6	81.8
	Fill volume of balloon	66.7	92.9	69.6	84.8
	Duration of balloon implantation (when removed)	75.0	89.3	87.5	94.1
	Height of staples used	58.6	84.4	48.0	64.7*
	Distance between resection and pylorus (for sleeve gastrectomy only)	82.8	83.9	76.0	82.4
	Size of bougie	79.3	96.9	76.0	76.5
	Hiatus hernia repair undertaken	82.8	96.9	92.0	85.3
	Measurements of limb length (not for sleeve gastrectomy)	93.1	96.8	96.0	100.0
	Pre-operative assessment of surgical risk, e.g., OS-MRS score or similar	75.9	93.8	72.0	82.4
	Name of surgical procedure, e.g., sleeve gastrectomy, one-anastomosis gastric bypass	96.6	93.8	100.0	100.0
	Surgical approach to gain access, e.g., laparoscopic, open or endoscopic	93.1	93.8	92.0	97.1
	Closure of potential internal hernia defects undertaken (not for sleeve gastrectomy)	86.2	93.8	96.0	100.0
	Type of reinforcement used	53.6	77.4	52.0	70.6
3b (n = 25)	Vitamin and mineral levels	93.9	100.0	82.0	93.8
	Clinical malnutrition	93.9	100.0	90.0	96.9
	Death from surgical complications whilst still in hospital (in-hospital mortality)	100.0	98.1	98.0	95.3
	Death after discharge from hospital (post-discharge mortality)	100.0	98.1	97.9	92.2
	Cause of death	98.0	98.1	89.6	92.2
	Obstruction including ileus and/or hernia (stapling/suturing procedures only)	97.8	98.0	95.5	95.3
	Problems with anastomotic/staple line/suture line including subsequent infections	95.7	98.0	95.5	96.9
	Complications that may occur shortly after the operation when the patient is still in hospital (device operations only)	97.8	96.1	95.6	96.9
	Complications that occur sometime after the operation, once the patient has been discharged (device operations only)	97.8	96.1	93.3	96.9
	Accidental damage to other organs (during surgery) (organ injury)	95.7	96.1	82.6	96.9
	Bleeding inside the body (intra-abdominal or endoluminal)	100.0	96.1	93.5	98.4
	Problems with the heart, vessels, or blood clots (cardiovascular problems or venous thromboembolism)	95.7	96.1	93.3	98.4
	Unplanned use of high dependency, intensive care or critical care units	95.7	96.1	88.9	92.2
	Whether a re-intervention occurred, including a classification of its severity, e.g., Clavien-Dindo	95.7	96.0	93.0	100.0
	Problems with the kidneys, including rhabdomyolysis (renal problems)	93.5	94.1	86.7	93.8
	Problems swallowing or bringing food back up (dysphagia/regurgitation)	83.7	92.3	77.1	90.6
	Problems with gastric and/or stomal ulcers	89.4	92.2	91.5	95.3
	Problems with drops in blood sugar after a meal (reactive hypoglycaemia)	81.6	92.2	72.3	84.4
	The amount and type of food patients consume (nutritional intake)	81.6	90.4	66.0*	85.9
	Liver problems	91.5	90.0	84.8	92.2
	Pain/discomfort in the body	72.3	82.7	54.0*	73.4
	Food moving too quickly from the stomach into the small intestine causing symptoms such as cramps, diarrhoea, nausea, feeling hot and sweaty (dumping syndrome)	81.6	82.7	71.4	87.5
	Problems with gallstones	71.4	82.7	63.0*	79.7
	Problems with bone strength (bone density)	70.2	78.4	54.3*	70.3
	Feeling sick or vomiting (nausea)	63.3	74.0	51.0	75.4
	***Number of items prioritised***	***58***	***70***	***53***	***66***

* denotes a discordant item (an item that only one group prioritised)

^1^ A grey cell indicates an item that has changed from ‘not prioritised’ to ‘prioritised’ or vice versa between rounds 1 and 2

^2^ A green cell indicates an item that was not prioritised in round 1 but was in round 2

^3^ A pink cell indicates an item prioritised in round 1 but not round 2

### Agreement of items prioritised

In rounds 1 and 2 respectively, 86 (89%) and 93 (96%) items were concordant (prioritised or not prioritised by both groups) and 11 (11%) and 4 (4%) were discordant (prioritised or not prioritised by only one group) (Table 3).

**Table 3:** Agreement between groups in prioritised items.

Core set number (number of items)	Round	Number of items prioritised^1^ by…				% discordant items	% agreement	Cohen’s kappa
		Both groups	Standard group only	Enhanced group only	Neither group			
***Core set 1 (n = 15)***	*Round 1*	5	3	0	7	20	80	κ =0.61^****^
	*Round 2*	6	2	0	8	13	87	κ =0.74^****^
***Core set 2 (n = 33)***	*Round 1*	15	1	3	14	12	88	κ =0.76^****^
	*Round 2*	22	^9^1	0	10	3	97	κ =0.93^*****^
***Core set 3a (n = 17)***	*Round 1*	10	0	0	7	0	100	κ =1.00^*****^
	*Round 2*	13	1	0	3	6	94	κ =0.82^*****^
***Core set 3b (n = 32)***	*Round 1*	20	4	0	8	12	88	κ =0.71^****^
	*Round 2*	25	0	0	7	0	100	κ =1.00^*****^
***Total (n = 97)***	*Round 1*	***50***	***8***	***3***	***36***	***11***	***89***	***κ =0.77*** ^****^
	*Round 2*	***66***	***4***	***0***	***28***	***4***	***96***	***κ =0.90*** ^*****^

^1^scored 7–9 by ≥70% of participants

**** substantial agreement (κ 0.61–0.80)

***** almost perfect or perfect agreement (κ 0.81–1.0)

In round 1, agreement between groups in prioritised items was ‘substantial’ for core sets 1, 2 and 3b (κ = 0.61, 0.76 and 0.71, respectively) and perfect (κ = 1.0) for core set 3a (Table 3). The overall agreement for the four core sets was ‘substantial’ (κ = 0.77). In round 1, the standard instructions group prioritised 58 items and the enhanced instructions group 53, although there were 11 discordant items (three in core set 1, four in core set 2 and four in core set 3b) (Tables 2 and 3).

In round 2, agreement was found to be substantial for core set 1 (κ = 0.74), almost perfect for core sets 2 and 3a (κ = 0.93 and 0.82 respectively) and perfect for core set 3b (κ = 1.0). The overall agreement for the four core sets combined was κ = 0.90 indicating almost perfect agreement. In round 2, the standard instructions group prioritised 70 items and the enhanced instructions group 66 items. These 66 items were common to both instruction groups. The four discordant items prioritised by the standard instructions group were ‘details of the MDT’ (core set 1), ‘pre-surgery

weight loss’ (core set 1), ‘how well the pancreas produces insulin’ (core set 2) and ‘height of staples used’ (core set 3b) (Tables 2 and 3).

Overall, agreement between groups in prioritised items improved in round 2 for three of the four core sets. Overall agreement was higher in round 2 than round 1 (κ = 0.9 versus κ = 0.77) with only 4% discordant items, compared to 11% in round 1 (Table 3).

### Differences in prioritisation of ‘Top 5’ items

The differences between groups in the percentage of participants prioritising round 1 ‘Top 5’ items are shown in Table 4. Differences between rounds 1 and 2 in the percentages of participants receiving standard instructions who prioritised the Top 5 items ranged from −2.8% (name of surgical procedure 96.6% to 93.8%) to +14.1% (hiatus hernia repair 82.8% to 96.9%). The mean change across the four core sets was + 2.3%. In the enhanced instructions group, differences ranged from −6.7% (hiatus hernia 92.0% to 85.3%) to +20.5% (other medical conditions not directly related to obesity 64.1% to 84.6%), with a mean change across the four core sets of +3.2%.

**Table 4:** Differences between groups in the percentage of participants prioritising round 1 ‘Top 5’ items.

Core set	Top 5 items within each core set from Round 1	Standard instructions			Enhanced instructions			Difference between standard and enhanced^1^
		Round 1 (% scoring 7–9)	Round 2 (% scoring 7–9)	Difference (%)^1^	Round 1 (% scoring 7–9)	Round 2 (% scoring 7–9)	Difference (%)^1^	
1	History of previous types of bariatric surgery	96.5	96.5	0	96.8	95.4	−1.4	−1.4
	Duration of type 2 diabetes	89.5	93.0	+3.5	73.4	83.1	+9.7	+6.2
	Age of the patient	82.5	80.7	−1.8	85.9	87.7	+1.8	+3.6
	Height of the patient	82.5	86.0	+3.5	77.8	90.8	+13.0	+9.5
	Other medical conditions not directly related to obesity	80.7	84.2	+3.5	64.1	84.6	+20.5	+17.0
**2**	Weight	96.2	96.3	+0.1	96.2	96.9	+0.7	+0.6
	Type 2 diabetes status	94.4	98.1	+3.7	96.4	96.9	+0.5	−3.2
	Medication for type 2 diabetes	92.6	96.3	+3.7	92.6	92.2	−0.4	−4.1
	Sleep apnoea	88.5	92.6	+4.1	88.7	95.3	+6.6	+2.5
	GERD	84.9	92.5	+7.6	83.3	95.3	+12.0	+4.4
**3a**	Name of surgical procedure	96.6	93.8	−2.8	100.0	100.0	0	+2.8
	Surgical approach to gain access	93.1	93.8	+0.7	92.0	97.1	+5.1	+4.4
	Closure of potential internal hernia defects	86.2	93.8	+7.6	96.0	100.0	+4.0	−3.6
	Measurements of limb length	93.1	96.8	+3.7	96.0	100.0	+4.0	+0.3
	Hiatus hernia repair	82.8	96.9	+14.1	92.0	85.3	−6.7	−20.8
**3b**	Death from surgical complications whilst still in hospital	100.0	98.1	−1.9	98.0	95.3	−2.7	−0.8
	Obstruction including ileus and/or hernia	97.8	98.0	+0.2	95.5	95.3	−0.2	−0.4
	Complications occur after operation when patient still in hospital	97.8	96.1	−1.7	95.6	96.9	+1.3	+3.0
	Death after discharge	100.0	98.1	−1.9	97.9	92.2	−5.7	−3.8
	Cause of death	98.0	98.1	+0.1	89.6	92.2	+2.6	+2.5
			**Mean change**	+**2.3**			**+3.2**	**+0.9**

^1^Change in percentage of participants prioritising item, illustrated as percentage change from round

## Discussion

This study examined the effects of providing summarised feedback on the prioritisation of items during a Delphi survey to reach consensus on the items to be included in a core data set. It was an exploratory randomised design, within the confines of an already planned core set development study, and therefore not powered sufficiently, so findings should be interpreted with this in mind. Results indicated that, overall, enhancing participant instructions in round 2 by including information about which five items were rated the highest in round 1, does not further promote consensus. Rather, both instruction groups prioritised a larger number of items in round 2 than in round 1 and there was little difference in the percentage of respondents prioritising the ‘Top 5’ items in round 2. Additional survey rounds due to lack of prioritisation and subsequent loss of participants due to survey fatigue may diminish the rigor of the core sets. Core sets are a useful tool in standardising research outputs, and gaining consensus is at the heart of this process.

Growing interest in core set methodology suggests that core set developers are trying to establish the best methodology to facilitate prioritisation and consensus. In the last five years (2019-current) [7], 64 studies were registered with the COMET database as investigating ‘COS methods research’. These studies explore numerous aspects of core set development including the ordering of survey items (e.g., patient-reported before/after clinical outcomes) [20], Likert rating scales [21,22] and presentation of feedback to participants in subsequent round(s) [6]. However, currently, there is no definitive method for developing COSs. Guidance documents created to standardise the development and reporting of a COS [3,7,15,16] navigate users through the process but do not prescribe methods for accomplishing COS generation and researchers continue to take different approaches [4]. The COS-STAP checklist states that the definition of consensus and how patients will receive feedback during the consensus process should be described [3], but does not provide guidance about developing survey and/or prioritisation instructions. This study has identified research questions about the role of instructions to achieve consensus during core set development but also highlights other areas of the Delphi process that warrant further exploration.

We hypothesised that the use of enhanced instructions, which provided summarised feedback in the form of the top five items prioritised by participants in round 1 for each core set, would efficiently guide participants through a sea of ‘critically important’ health items to those voted ‘critically important’ by the greatest proportion of participants. We reasoned that participants would be in a more informed position to re-rate the top five items higher, if in agreement, or lower, to indicate disagreement, thereby promoting fewer items being prioritised and consensus reached on a minimal core set. Instead, however, we observed that a greater number of items were prioritised, making the finalisation of a minimal core set, more challenging. The absence of prioritisation in both groups suggests that the presentation of median scores with or without a ‘Top 5’ did not facilitate prioritisation further and the reasons for this need to be considered. It is plausible that, in an effort to improve *consensus* (by modifying their opinions more in line with others’), stakeholders overlooked the aim of *prioritising* only ‘critically important’ items, by scoring more items as ‘important’. This finding highlights complexities in the mechanisms by which instructions and feedback influence participants’ behaviour in Delphi surveys, suggesting the need for in-depth qualitative work to explore participants’ interpretations and understanding during core set development. Indeed, it may have been a valuable addition to this work to have interviewed participants following completion of the surveys, however it was outside the scope of the study. Such work may focus on improving participants’ understanding of the concepts of ‘prioritisation’ and ‘consensus’ and how instructions may optimally distinguish between the two while simultaneously promoting both.

Previous qualitative work has explored participants’ understanding of the purpose of a COS and the Delphi process [23,24]. Through online feedback surveys [24] and in-person interviews [23], it was evident that participants’ understanding was variable [23,24] and was affected by previous experience with COS development and/or the Delphi survey process [23]. It was concluded that participants would benefit from repeated guidance on the principles for COS development during voting [24] and that further guidance and support needed to be accessible and salient [23]. Whilst the COMET Initiative website provides resources for lay audiences to explain the concepts of COSs and Delphi consensus process [25], these lack the detail required to differentiate between prioritisation and consensus. Understanding the relationship between these two concepts is important in finalising a minimum core set in a timely manner. Biggane’s recommendation [23] of considering the most appropriate medium(s) to communicate the core set study is pertinent. Whilst written instructions have historically been the norm and are undoubtable the simplest and quickest form of communication, they are perhaps not the most engaging and may even be ambiguous to readers. Use of more visual resources [23] such as demonstration videos [26] may help in engaging and educating participants. A novel ‘live’ approach in COS development was recently published [27]; researchers ran a Delphi ‘hackathon’ where all participants simultaneously completed the online Delphi surveys [27]. This live event involved an introductory session explaining the methodology and purpose of the study and provided breakout rooms for any questions regarding any aspect of the process [27]. Subsequent rounds emphasised that the goal of the Delphi survey was to reach consensus and participants were able to ask questions via a chat function [27]. The ability for participants to access timely guidance and support is undoubtedly valuable, and something which is not possible with current Delphi survey methods. It is vitally important that those taking part in a Delphi survey understand the significance of the role of feedback since utilising feedback to reconsider opinions and responses in order to gain consensus is fundamental to this process. If participants are not using the provided feedback effectively, then the process is not fit for purpose. Further qualitative work to explore how to clarify and communicate the concepts of prioritisation and consensus to all stakeholder groups in Delphi instructions, whether written, verbal or visual, is needed.

In addition to improving communication, ways by which to expedite prioritisation could be explored. The James Lind Alliance (JLA) specialises in prioritising research questions to be answered in specific healthcare fields [28,29]. The methodology involves asking relevant stakeholders what are the most important questions to research, collating this information and then asking stakeholders to rank or choose their top 10 research questions to be taken to a workshop for further discussion^30^ – a process very similar to core set development using Delphi techniques. As with all methodologies, there are advantages and disadvantages (as described in their guidebook [30]), but ultimately the outcome is a prioritised set of data items.

A strength of this study was that participants were not aware of their allocation to receive different instructions in the second survey round and responses were therefore free from performance bias. This study used a randomised design, in which participants who were assigned an identity number in the first survey round, were randomised by the REDCap software [11,12], to receive standard or enhanced instructions in the second survey round. Whilst randomisation ensures balanced groups, a potential limitation of using REDCap software to perform randomisation in this study was that all identity numbers were assigned to standard or enhanced feedback, regardless of whether participants answered questions or opened the survey numerous times. By including participants with multiple identity numbers and non-responders, the randomisation of participants to the two groups was not equal. This could have resulted in selection bias, however, in this study, baseline characteristics were similar between both groups and allocation did not affect response rates. Though desirable, not all nested COS methodology studies are randomised [21,31]. In addition, because this nested study was opportunistic in nature, it was not powered to be able to detect meaningful differences between the randomised groups and as such, results should be interpreted with caution and need further validation. A larger sample size would have increased the confidence of detecting a true effect of the different instruction types between the two randomisation groups and would have allowed more in-depth statistical analyses of the effects on individual stakeholder groups. When working within study limitations, efforts to engage and retain participants should be prioritised to increase the sample size and improve the strength and validity of the study. Response rates in round 2 were below half, lower than our previous core sets [13,17,32,33]. A participant was considered to have ‘responded’ if at least one core data item question had been answered. In round 1, this would likely have resulted in the inclusion of individuals curious about the survey content but then choosing not to proceed beyond the first few questions or first section (almost a quarter of participants did not continue to section 2). Ultimately, this would have led to inflated response rates in round 1 and exaggerated attrition rates in round 2. Comprehensive datasets strengthen the validity of study findings since missing data can introduce bias depending on the reason for omission. Omission, and therefore attrition, can be attributed to a number of factors including the length of the survey and the time elapsed between the first and final round [4], but in this instance could also be attributed to the inclusion of non-invested individuals. Ultimately, there is no guidance as to the number of completed questions required for inclusion as a ‘respondent’ or the length of the survey or the duration of the core set development process. But further work to explore completion inclusion thresholds and methods to promote completion and thereby reduce attrition would all be beneficial in promoting a comprehensive dataset. Finally, a key reflection in the writing up of this study is the recognition of the role that qualitative work would have played in progressing our understanding of how feedback was interpreted and applied.

## Conclusion

Using a nested study approach within the development of a registry core data set to explore core set methodology, this study suggests that enhanced instructions emphasising the top items prioritised do not promote the prioritisation of items in a Delphi survey during core set development. Due to the complexities of the core set development process, it is likely that there are multiple factors at play. The absence of prioritisation in both groups perhaps suggests a lack of, or limited, understanding of the pathway to gaining consensus. Further work to explore how participants interpret and respond to feedback and instructions to simultaneously prioritise items and reach consensus with others is warranted. Educating and communicating these crucial elements will improve the efficiency and the value of the core set.

## Supporting information

S1 Table Proportion of stakeholders scoring items as ‘critically important’ (score 7–9). (DOCX)
