# Supplementary material for: Optimising the Delphi survey method during core set development: The impact of summarised feedback on stakeholders’ prioritisation of core data items and consensus
Source: PLoS One. 2026 Jun 10;21(6):e0348136. doi: 10.1371/journal.pone.0348136 (PMC13252763; doi:10.1371/journal.pone.0348136)
Supplement: S1 Table — (DOCX) [file pone.0348136.s001.docx]

**Supplementary Table 1.** Proportion of stakeholders scoring items as ‘critically important’ (score 7-9)

|  |  | **Standard instructions (% scoring 7-9)** | | **Enhanced instructions (% scoring 7-9)** | |
| --- | --- | --- | --- | --- | --- |
|  |  |  |  |  |  |
| **Core set** | **Item (n=97)** | **Round 1** | **Round 2** | **Round 1** | **Round 2** |
| **1 - Baseline information** | Source of funding to pay for the surgery, e.g., private health care insurance, public funding/national health service, self-paying | 46.4 | 57.9 | 46.8 | 52.3 |
|  | Date on which the patient was referred for surgery | 49.1 | 43.9 | 38.1 | 28.1 |
|  | Details of what members of the multi-disciplinary team have been involved with the patient to date | 70.2 | 71.4 | 66.2 | 69.2 |
|  | Sex of the patient | 71.9 | 77.2 | 71.9 | 76.9 |
|  | Age of the patient | 82.5 | 80.7 | 85.9 | 87.7 |
|  | Ethnicity of the patient | 46.4 | 45.6 | 51.6 | 44.6 |
|  | Educational level of the patient | 33.3 | 33.3 | 46.9 | 44.6 |
|  | Ability of patient to purchase/afford supplements for life, post-surgery | 50.0 | 60.7 | 53.1 | 60.9 |
|  | Height of the patient | 82.5 | 86.0 | 77.8 | 90.8 |
|  | History of previous types of bariatric surgery | 96.5 | 96.5 | 96.8 | 95.4 |
|  | Details of previous weight loss programs | 70.2 | 69.6 | 58.7 | 56.9 |
|  | Details about pre-surgery weight loss | 68.4 | 75.0 | 66.7 | 67.7 |
|  | Time period over which pre-surgery weight loss occurred | 49.1 | 57.9 | 47.5 | 45.3 |
|  | Duration of type 2 diabetes | 89.5 | 93.0 | 73.4 | 83.1 |
|  | Other medical conditions not directly related to obesity e.g., type 1 diabetes, organ transplantation, dementia | 80.7 | 84.2 | 64.1 | 84.6 |
|  |  | | | | |
| **2 - Effectiveness outcomes** | Abnormal or irregular heartbeat, or use of medication (arrhythmia) | 53.1 | 79.6 | 69.1 | 76.6 |
|  | High blood pressure, or use of medication (hypertension) | 76.5 | 88.9 | 80.4 | 89.1 |
|  | Risk of future heart and vascular problems (assessment of cardiovascular risk) | 74.5 | 79.2 | 59.3 | 81.3 |
|  | Congestive heart failure, or use of medication | 78.0 | 92.5 | 71.2 | 82.8 |
|  | Type 2 diabetes status | 94.4 | 98.1 | 96.4 | 96.9 |
|  | Medication for Type 2 diabetes | 92.6 | 96.3 | 92.6 | 92.2 |
|  | How well the pancreas produces insulin (ß-cell function) | 59.2 | 73.6 | 49.0 | 54.0 |
|  | Elevated fat and cholesterol in the blood, or use of medication (dyslipidemia) | 79.2 | 85.2 | 73.1 | 81.3 |
|  | Problems with breathing during sleep (obstructive sleep apnoea) | 88.5 | 92.6 | 88.7 | 95.3 |
|  | Ability to fall asleep at night or quality of sleep (sleep disorders other than sleep apnoea) | 36.5 | 61.1 | 48.1 | 63.1 |
|  | Joint disease, or use of medication, or being considered for joint replacement | 64.7 | 70.4 | 71.7 | 78.1 |
|  | Long standing acid reflux, or use of medication (gastro-esophageal reflux or GERD) | 84.9 | 92.5 | 83.3 | 95.3 |
|  | Bladder problems (urinary incontinence) | 34.7 | 30.2 | 31.4 | 26.6 |
|  | Long standing diseases of the lungs such as asthma (chronic pulmonary disease) | 55.1 | 66.7 | 56.9 | 62.5 |
|  | Thyroid function, or use of medication (hypothyroidism) | 42.3 | 59.3 | 53.8 | 63.5 |
|  | Obesity-related liver disease, e.g., non-alcoholic fatty liver disease | 75.5 | 86.8 | 81.1 | 90.6 |
|  | Male or female reproductive function, e.g., polycystic ovary syndrome, infertility (reproductive dysfunction) | 66.0 | 77.8 | 71.2 | 73.8 |
|  | Long standing fluid retention (lymphedema) | 34.0 | 50.9 | 33.3 | 32.8 |
|  | Abnormal accumulation of fat in legs/arms (lipedema) | 36.0 | 41.5 | 32.7 | 31.3 |
|  | Suicidal thoughts | 80.8 | 92.6 | 72.7 | 89.2 |
|  | Binge eating | 86.5 | 92.6 | 85.2 | 87.7 |
|  | Depression, or use of medication | 78.8 | 92.6 | 79.6 | 92.3 |
|  | Feelings towards one's body shape or appearance (body dysmorphia/dysmorphic disorder) | 68.6 | 83.3 | 59.3 | 73.8 |
|  | Addictive behaviours, e.g., alcohol, gambling, illicit drugs | 76.9 | 96.3 | 76.4 | 92.3 |
|  | Anger management problems | 34.7 | 44.4 | 32.0 | 33.8 |
|  | Weight | 96.2 | 96.3 | 96.2 | 96.9 |
|  | Body shape, e.g., waist and hip measurements | 57.7 | 72.2 | 59.6 | 70.3 |
|  | Alcohol intake | 81.6 | 92.6 | 75.0 | 90.8 |
|  | Smoking status | 72.0 | 90.7 | 76.9 | 90.6 |
|  | Employment status | 49.0 | 46.3 | 35.3 | 31.3 |
|  | Family and relationship status | 63.3 | 61.1 | 39.2 | 50.8 |
|  | Gut microbiota (gut flora) | 37.8 | 30.8 | 35.6 | 36.5 |
|  | Use of weight loss medication | 67.3 | 77.4 | 80.4 | 73.4 |
| **3a - Surgical procedure information** | Pre-operative assessment of surgical risk, e.g., OS-MRS score or similar | 75.9 | 93.8 | 72.0 | 82.4 |
|  | Length of time spent on the waiting list for surgery | 48.3 | 34.4 | 40.0 | 32.4 |
|  | Length of time spent in hospital after admission for surgery | 51.7 | 68.8 | 48.0 | 64.7 |
|  | Name of surgical procedure, e.g., sleeve gastrectomy, one-anastomosis gastric bypass | 96.6 | 93.8 | 100.0 | 100.0 |
|  | Surgical approach to gain access, e.g., laparoscopic, open or endoscopic | 93.1 | 93.8 | 92.0 | 97.1 |
|  | Height of staples used | 58.6 | 84.4 | 48.0 | 64.7 |
|  | Make of stapler used | 51.7 | 67.7 | 37.5 | 52.9 |
|  | Type of reinforcement used | 53.6 | 77.4 | 52.0 | 70.6 |
|  | Size of bougie | 79.3 | 96.9 | 76.0 | 76.5 |
|  | Distance between resection and pylorus (for sleeve gastrectomy only) | 82.8 | 83.9 | 76.0 | 82.4 |
|  | Hiatus hernia repair undertaken | 82.8 | 96.9 | 92.0 | 85.3 |
|  | Closure of potential internal hernia defects undertaken (not for sleeve gastrectomy) | 86.2 | 93.8 | 96.0 | 100.0 |
|  | Measurements of limb length (not for sleeve gastrectomy) | 93.1 | 96.8 | 96.0 | 100.0 |
|  | Type/make of device (including band and balloon, adjustable or non-adjustable) | 76.9 | 86.2 | 72.0 | 88.2 |
|  | Method of balloon placement, e.g., swallowed or endoscopically placed | 66.7 | 85.7 | 69.6 | 81.8 |
|  | Fill volume of balloon | 66.7 | 92.9 | 69.6 | 84.8 |
|  | Duration of balloon implantation (when removed) | 75.0 | 89.3 | 87.5 | 94.1 |
| **3b - Potential complications and side effects of surgery** | Death from surgical complications whilst still in hospital (in-hospital mortality) | 100.0 | 98.1 | 98.0 | 95.3 |
|  | Death after discharge from hospital (post-discharge mortality) | 100.0 | 98.1 | 97.9 | 92.2 |
|  | Cause of death | 98.0 | 98.1 | 89.6 | 92.2 |
|  | Problems with anastomotic/staple line/suture line including subsequent infections | 95.7 | 98.0 | 95.5 | 96.9 |
|  | Obstruction including ileus and/or hernia (stapling/suturing procedures only) | 97.8 | 98.0 | 95.5 | 95.3 |
|  | Complications that may occur shortly after the operation when the patient is still in hospital (device operations only) | 97.8 | 96.1 | 95.6 | 96.9 |
|  | Complications that occur sometime after the operation, once the patient has been discharged (device operations only) | 97.8 | 96.1 | 93.3 | 96.9 |
|  | Accidental damage to other organs (during surgery) (organ injury) | 95.7 | 96.1 | 82.6 | 96.9 |
|  | Bleeding inside the body (intra-abdominal or endoluminal) | 100.0 | 96.1 | 93.5 | 98.4 |
|  | Problems with the heart, vessels, or blood clots (cardiovascular problems or venous thromboembolism) | 95.7 | 96.1 | 93.3 | 98.4 |
|  | Problems with the kidneys, including rhabdomyolysis (renal problems) | 93.5 | 94.1 | 86.7 | 93.8 |
|  | Problems with gastric and/or stomal ulcers | 89.4 | 92.2 | 91.5 | 95.3 |
|  | Unplanned use of high dependency, intensive care or critical care units | 95.7 | 96.1 | 88.9 | 92.2 |
|  | Liver problems | 91.5 | 90.0 | 84.8 | 92.2 |
|  | Feeling sick or vomiting (nausea) | 63.3 | 74.0 | 51.0 | 75.4 |
|  | Whether a re-intervention occurred, including a classification of its severity, e.g., Clavien-Dindo or similar | 95.7 | 96.0 | 93.0 | 100.0 |
|  | Pain/discomfort in the body | 72.3 | 82.7 | 54.0 | 73.4 |
|  | Problems with bowel movements/flatulence | 60.4 | 69.2 | 45.8 | 60.9 |
|  | Problems swallowing or bringing food back up (dysphagia/regurgitation) | 83.7 | 92.3 | 77.1 | 90.6 |
|  | Skin problems or irritations, e.g., rashes, sores, loose skin, or ulcers or exacerbation of existing skin problems | 45.8 | 44.2 | 39.1 | 43.8 |
|  | Food moving too quickly from the stomach into the small intestine causing symptoms such as cramps, diarrhea, nausea, feeling hot and sweaty (dumping syndrome) | 81.6 | 82.7 | 71.4 | 87.5 |
|  | Problems with gallstones | 71.4 | 82.7 | 63.0 | 79.7 |
|  | Problems with drops in blood sugar after a meal (reactive hypoglycaemia) | 81.6 | 92.2 | 72.3 | 84.4 |
|  | Problems with bone strength (bone density) | 70.2 | 78.4 | 54.3 | 70.3 |
|  | Problems with teeth | 59.6 | 64.0 | 40.4 | 48.4 |
|  | Hair loss | 51.0 | 56.9 | 44.0 | 53.1 |
|  | Problems with kidney stones | 51.1 | 49.0 | 35.6 | 48.4 |
|  | Leg cramps | 38.6 | 29.4 | 26.7 | 21.9 |
|  | Problems with immune system, e.g., recurrent infections | 38.6 | 35.3 | 37.8 | 40.6 |
|  | The amount and type of food patients consume (nutritional intake) | 81.6 | 90.4 | 66.0 | 85.9 |
|  | Vitamin and mineral levels | 93.9 | 100.0 | 82.0 | 93.8 |
|  | Clinical malnutrition | 93.9 | 100.0 | 90.0 | 96.9 |
